# Oral Pirfenidone in patients with chronic fibrosis resulting from radiotherapy: a pilot study

**DOI:** 10.1186/1748-717X-2-19

**Published:** 2007-05-31

**Authors:** Nicole L Simone, Benjamin P Soule, Lynn Gerber, Elizabeth Augustine, Sharon Smith, Rosemary M Altemus, James B Mitchell, Kevin A Camphausen

**Affiliations:** 1 Radiation Oncology Branch, National Cancer Institute, National Institutes of Health, 9000 Rockville Pike, Building 10-CRC, Room B2-3561, Bethesda, Maryland, 20892, USA; 2 Radiation Biology Branch, National Cancer Institute, National Institutes of Health,9000 Rockville Pike, Building 10, Room B3B69, Bethesda, Maryland, 20892, USA; 3 Center for Chronic Illness and Disability, Department of Global and Community Health, George Mason University, Robinson B415B, Mail Stop 5B7, 4400 University Drive, Fairfax, Virginia, 22030, USA

## Abstract

**Background:**

Fibrosis is a common side effect after treatment with ionizing radiation. Several methods to ameliorate debilitating fibrosis have been employed but without consistent results. The goal of this pilot study is to determine if Pirfenidone, a novel regulator of cytokine gene expression, has the potential to ameliorate established radiation-induced fibrosis.

**Methods:**

Open label, prospective pilot study of 800 mg three times/day, orally administered Pirfenidone was administered to enrolled patients who were had completed radiation therapy and who had established radiation-induced fibrosis. Range of motion (ROM) was assessed using standard measures, and subjective measures of pain, fatigue, disability and global health were measured every three months.

**Results:**

Seven patients were enrolled of whom 3 had ROM assessments of 1 site and 2 had ROM assessments of 2 sites. Of these assessments, 6 revealed increased ROM during drug intervention while 1 revealed a decreased ROM. There was an overall improvement in the mental composite score of the SF36 while physical composite score was decreased and the vitality score was unchanged. Two patients were removed from the study because of syncopal episodes.

**Conclusion:**

Several patients experienced improved function of at least 25% and reported subjective improvement. Pirfenidone may benefit patients with radiation-induced fibrosis and is worthy of a larger well controlled trial.

## Background

Fibrosis is a major cause of morbidity after treatment with ionizing radiation [1-3]. Manifestations of fibrosis are usually tissue specific with effects ranging from limitation of mobility to poor wound healing and neuropathy. For example, irradiation of the brain induces gliosis whereas in the lung, radiation results in an acute inflammatory response often followed by chronic fibrosis manifested as restrictive lung disease and decreased diffusion capacity. Radiation-induced fibrosis of the skin and connective tissue can lead to contractures, ulcerations and neuropathy resulting in decreased mobility and difficulty swallowing and speaking [4]. As a result, fibrosis is a dose-limiting toxicity related to radiotherapy.

While the mechanism underlying chronic fibrosis is not fully known, vascular damage and the release of inflammatory cytokines and other factors such as platelet derived growth factor (PDGF), endothelial growth factor (EGF) and fibroblast growth factor (FGF) stimulate fibroblast activation which seems to initiate a pro-fibrotic cascade of events [5-10]. Decreasing the radiation dose to sensitive structures is the mainstay of prevention of fibrosis. Other approaches moderate the developing fibrotic process post-irradiation including administration of hyperbaric oxygen, and the use of drugs such as pentoxifylline [11] and Vitamin E [12]. Recent studies report the potential benefit of combined therapy using Vitamin E and pentoxifylline after radiotherapy to reverse fibrosis [13,14] but larger trials are needed to confirm these results.

Pirfenidone (5-methyl-N-phenyl-2-(1H)-pyridone) is a novel, small, non-peptide, selective regulator of gene expression induced by molecular signals from cytokines such as transforming growth factor-β1 (TGF-β1), PDGF, β-FGF, EGF, tumor necrosis factor-α(TNF-α) and related families. Pirfenidone has been shown in several animal models as well as human *in vitro *studies to alter cytokine signaling and reduce or eliminate pulmonary inflammation and fibrosis. Pirfenidone is currently being evaluated in phase 2 and 3 clinical trials for the treatment of idiopathic pulmonary fibrosis [15] and trials are ongoing for patients with scleroderma, sclerosing peritonitis, proliferative vitreoretinopathy, myelofibrosis with myeloid metaplasia, renal focal segmental glomerulosclerosis and patients with pulmonary fibrosis associated with Hermansky-Pudlak Syndrome (a list of trials involving Pirfenidone is available at ). No studies have been conducted, however, looking at the effectiveness of Pirfenidone in ameliorating or treating established fibrosis resulting from radiation therapy. We initiated this pilot study to examine whether Pirfenidone, administered in a daily oral dose, can decrease chronic radiation-induced fibrosis and lead to improvements in mobility and function.

## Methods

The patients enrolled in this IRB approved study from our follow-up clinic had undergone regional radiation treatments and had developed fibrosis of the neck, back or extremities that caused at least moderately severe loss of range of motion (> 25% normal range of motion), strength, swallowing or significant edema. Patients had completed radiation therapy at least six months prior to enrollment to ensure that observations were not confounded by acute radiation effects. The patients were given a total of 2400 mg Pirfenidone orally each day divided into three 800 mg doses which is the same dose used in other trials studying the efficacy of Pirfenidone [16,17]. The patients were seen in our radiation oncology follow-up clinic as well as in the Rehabilitation Medicine Department every three months while receiving Pirfenidone. Prior to initiating therapy and at each subsequent visit, the patients were questioned about subjective changes in fibrosis-related symptoms and potential adverse effects related to the medication. Compliance with therapy was determined by patient self-reporting as well as pill counts at each visit. Patients were encouraged to call the treating physician immediately if they developed any new symptoms or problems.

Enrolled patients were evaluated at baseline and at three monthly intervals by an experienced and trained rehabilitation staff who performed active and passive range of motion assessments, muscle testing and administered questionnaires as per the protocol. Cervical range of motion, using an inclinometer, was measured in 4 planes of motion including flexion, extension, right and left rotation and right and left lateral flexion. Ten upper and lower extremity ranges of motion were measured including extension, flexion, adduction, abduction, internal rotation, external rotation, extension and flexion at the elbow or knee, and extension and flexion at the wrist or ankle. For each body site, the maximum possible ROM was calculated by summing the degrees of freedom in all planes of motion. The patient's total ROM was determined by summing the actual measured degrees of ROM in each plane and this was compared to the maximum possible ROM. Finally, the patients completed five written surveys at each visit: the Fatigue Severity Scale (FSS) [18,19], the Human Activity Profile (HAP) [20], a measure of activity level, a Dyspnea scale [21], the Pain Disability Index (PDI) [22,23], and the SF-36 v.2 (Medical Outcomes Trust, Inc. Boston, MA) global health survey [24]. All of these are commonly employed, validated tools that reliably measure various patient reported factors related to health related quality of life and function. Patients were enrolled six months or longer after the completion of radiotherapy to eliminate potentially confounding acute inflammatory changes and were evaluated every three months for a total of two years. All patient data were collected and analyzed by the Rehabilitation Medicine Department.

## Results

**Figure 1:**
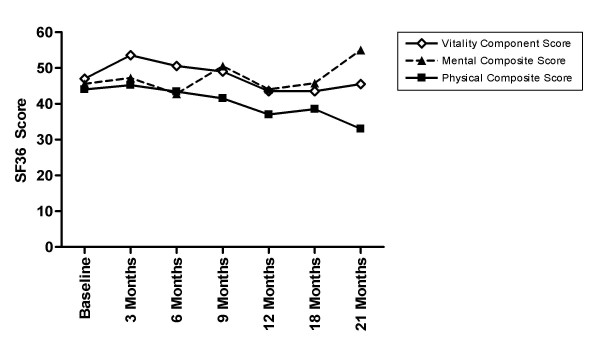
**Average SF36 Component Scores**. The average score for all patients for each component of the SF36 is shown. During drug therapy, the vitality component score shows little change (-3%), the physical composite score decreases by 33% while the mental composite score shows an increase of 17%.

Seven patients were enrolled in the study, however two were removed from the study after experiencing syncopal episodes during treatment and no further assessments of these patients were made. Of the five patients who remained in the study, all were treated for the entire two year period and their characteristics are noted in Table 1. The patients ranged in age from 53 to 60 years old and all were men. Four of the patients had been treated for head and neck cancer and one had been treated for Hodgkin's Disease. At the time of enrollment, 4 patients complained of limited cervical mobility of whom 2 also complained of limited UE mobility. One patient complained only of limited LE mobility. All of the patients reported a subjective improvement in their symptoms over time. This included a perceived improvement in their mobility and range of motion and subsequent improvement in activities of daily living. Analysis of the SF36 survey revealed a somewhat mixed picture. One patient demonstrated an improvement in the Vitality score, a subset of questions that measures energy and fatigue, while three remained unchanged and one had a decreased score. Two of the patients exhibited an improvement in the Mental Competency Composite Score (MCS), a component of the SF36 that reports a patient's impression of their overall health, however the other three had no change. In two cases there was a slight improvement in the Physical Competency Composite Score (PCS), a component of the SF36 that reports the patient's physical abilities and impairments, a significant decrease in one case and two patients reported no change. Taken as a group, there was an overall trend towards improvement in the MCS score of the SF36 which corresponds with the patient's subjective reports of improvement. There was, however, a slight overall worsening of the PCS and Vitality scores during the drug therapy (Figure 1). There were no significant changes in the Fatigue Severity Scale (FSS), the Human Activity Profile (HAP), the Dyspnea scale, or the Pain Disability Index (data not shown).

**Table 1:** Patient Characteristics

*Age*	*Gender*	*Tumor Location*	*Radiation Dose (Gy)*	*Time from Completion of Radiation (Months)*	*ROM Impairment at Baseline*	*Duration of Pirfenidone Treatment (Months)*
60	M	Head and Neck	63	46.0	Decreased Cervical ROM	25.0
60	M	Head and Neck	72	32.1	Decreased Cervical ROM	24.6
54	M	Hodgkin's	45 – Pelvis 36 – PA	198.8	Decreased LE ROM and Strength	24.0
59	M	Head and Neck	70.2	53.7	Decreased UE and Cervical ROM	23.3
56	M	Head and Neck	60	69.1	Decreased UE and Cervical ROM	22.4

53	M	Hodgkin's	54 – Mantle 36 – PA	276.5	Decreased Cervical ROM	6.3 (removed from study)
60	M	Head and Neck	70	23.4	Decreased Cervical ROM	3.0 (removed from study)

**Figure 2:**
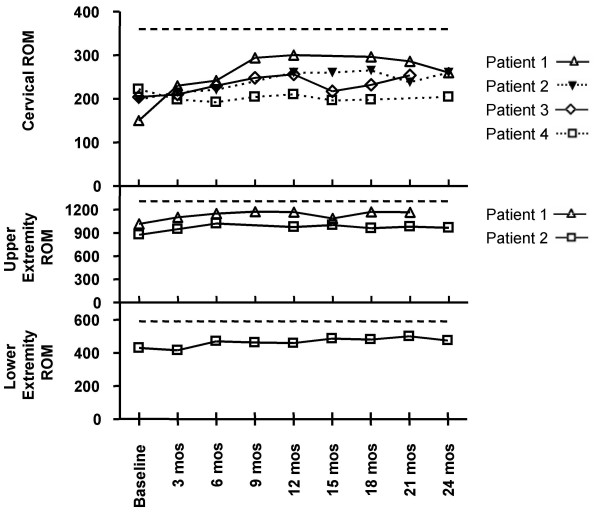
**Total Range of Motion Measurements**. The total ROM (sum for all planes of motion) was calculated for each patient for the cervical spine, upper extremity and lower extremity. Maximum possible total ROM is represented at the top of each graph by a dotted line. Each patient is represented individually. For the four patients with cervical ROM measurements, Patient 1 showed an improvement of 73%, Patient 2 improved by 31%, and Patient 3 improved by 24%. Patient 4 had a decline in function of 8%. Both patients with upper extremity ROM measurements show an improvement of over 100 degrees of ROM during the drug therapy with Patient 1 showing a 15% improvement and Patient 2 improving by 10%. For the single patient with lower extremity ROM measurements, there is a total improvement of approximately 50 degrees of ROM during the drug therapy which is 11% above baseline.

Like the SF36 MCS, the quantitative ROM measurements revealed a trend towards improvement. As previously described, the patient's total cervical spine ROM was measured in the four patients previously treated to the head and neck region (Figure 2). Of these, three had measurable improvement of 24, 31 and 73% while one had a decline in total ROM of 8%. Two patients had upper extremity measurements and both had an improvement of over 100 degrees total ROM translating to an improvement of 10 and 15% (Figure 2). The one patient who had lower extremity measurements showed an improvement in total ROM of nearly 50 degrees, or 11% above baseline (Figure 2).

Two patients discontinued the medication because of possible adverse events. Both patients had self-reported syncopal episodes while taking Pirfenidone and both underwent thorough evaluations by their primary care physicians and cardiologists but no definite etiology was found. Neither patient had suffered from a seizure, myocardial infarct, cerebrovascular accident or other vascular event. In both cases the patients recovered fully within minutes of the event and had no subsequent events that were reported to us. In the interest of patient safety, however, the events were reported to the manufacturer of the drug, and the patients were removed from the study.

## Discussion

This report documents the findings from a pilot study involving a small number of subjects treated with Pirfenidone, a novel regulator of cytokine gene expression, administered a minimum of six months after the completion of radiation therapy. Improvement in ROM was documented for six of the seven patients. In this study, patients exhibited a slight improvement in overall function as a result of treatment with Pirfenidone during the two year follow-up period. ROM was modestly but consistently improved over a two year period, and loss of range of motion over time is not uncommon in people of this age group. Additionally, the natural history of radiation induced fibrosis is that of slowly increasing loss of range of motion, with stiffness which may plateau but does not spontaneously improve. This is especially true for those with well established fibrosis and persistent functional loss. For all patients in this study, ROM improved and in several this was associated with functional improvement.

Fibrosis is a significant long-term side effect of treatment with ionizing radiation [1,2] which can limit mobility and contribute to poor wound healing and neuropathy [3]. Because of its frequency and potential severity, fibrosis can be a dose-limiting toxicity. Although the manifestations of fibrosis are tissue-specific, the underlying mechanism generally involves vascular damage and cytokine release [5-9]. Increased vascular permeability results in the influx of inflammatory cells and the subsequent release of factors such as TNF-α, TGF-β, and interleukins. These stimulate fibroblasts to increase production of extracellular matrix proteins and to secrete chemotactic signals that promote additional fibroblast recruitment resulting in a robust pro-fibrotic stimulatory cascade [25,26].

Several techniques to reduce fibrosis have been used with varying degrees of success but avoidance of irradiation remains the mainstay of prevention. Careful treatment planning allows for the irradiation of the smallest possible field at the lowest effective dose, however significant fibrosis is often inevitable. Several pharmaceuticals and the administration of hyperbaric oxygen have attempted to address the problem by targeting tissue hypoxia. However, the potential for the promotion of regrowth of residual malignant cells and the potentially toxic effects of oxygen itself have precluded the widespread use of hyperbaric oxygen, and most pharmaceutical agents have been found to be ineffective.

Administration of the drug pentoxifylline during or after radiation treatment is thought to improve microcirculation and oxygenation [11] and may prevent or even reverse the development of fibrosis. A recent review of the literature, however, found little evidence that pentoxifylline alone reduces the acute side effects of radiotherapy [27]. Many studies of pentoxifylline include the addition of Vitamin E analogues, but studies investigating the combination treatment have also had varying degrees of success [12]. An animal study compared the effect of pentoxifylline alone to the combination of pentoxifylline and α-tocopherol following exposure to a single 160 Gy radiation dose [28]. The authors reported a significant reduction in the formation of fibrosis with combination therapy but no benefit from pentoxifylline alone. A recent study found that four of 23 patients undergoing pelvic irradiation demonstrated significant clinical benefit from combined therapy with pentoxifylline and α-tocopherol, but none reported an improvement in symptoms or function on patient self-assessment questionnaires [13]. A study of 24 women who had undergone radiation therapy for breast cancer reported regression of fibrosis after 6 months of treatment with pentoxifylline and vitamin E [14]. No improvement was observed in patients taking either medication alone.

While several receptor tyrosine kinase inhibitors have been shown to block radiation-induced PDGF signaling resulting in decreased pulmonary fibrosis, no studies have been conducted examining the prevention of fibrosis at other sites [29]. Pirfenidone selectively regulates gene expression signaling from pro-fibrotic cytokines such as TGF-β1, PDGF, β-FGF, EGF, and TNF-α. In pre-clinical studies, Pirfenidone altered TGF-β transcription in a murine model of bleomycin-induced pulmonary fibrosis [30], IL-6 expression in rat models of acute pulmonary inflammation [31], and expression of ICAM-1 in cultured human fibroblasts [32]. Pirfenidone has also been shown to ameliorate cyclophosphamide and bleomycin-induced fibrosis in hamsters and mice [33,34], and liver fibrosis [35] and sclerosing peritonitis in rats [36]. This is the first study to test the possible usefulness of Pirfenidone in the treatment of radiation-induced deep tissue fibrosis.

Several metrics used in this study to evaluate the clinical response to Pirfenidone, such as questionnaires, are somewhat subjective. Although objective measures, such as standardized ROM measurements, were employed by physical therapists, some subjectivity remains because of the nature of the testing and because patients were not always assessed by the same therapist making subtle differences in patient function more difficult to detect.

In studies reporting on side effects associated with the use of Pirfenidone, gastrointestinal complaints were common [15,37], photosensitivity occurred in several patients [37,38] and in one study a single patient complained of dizziness [37]. In our cohort, most patients noted a phase-in period that was associated with fatigue and occasional nausea. These symptoms were mild and short lived and did not necessitate a dose adjustment. Nausea was improved when the medication was taken with food. Two patients in this trial had what appeared to be syncopal episodes. Both had significant fibrosis resulting from radiation treatment for head and neck cancer which can cause compression of the carotid sinus resulting in syncope and which may have been the underlying cause in these patients. Although these patients did not have any further episodes, they were removed from the study and the events were reported to the drug manufacturer. Syncope as a result of Pirfenidone treatment has not been reported elsewhere. No other significant side-effects were reported.

## Conclusion

The stabilization of function and fibrosis-related symptoms in this set of patients suggests that Pirfenidone may be effective in ameliorating the disability associated with radiation-induced fibrosis. Future trials are required to determine optimal therapeutic dosing, timing interval, duration of treatment, and to establish efficacy. Ideally, objective measures should include determination of tissue resilience using imaging technologies. Subjective measures utilizing a single blinded observer for ROM metrics should be of adequate sensitivity to document clinical meaningful change. Because other treatments are of limited utility, a larger, well controlled trial is warranted as Pirfenidone shows promise in ameliorating this debilitating side-effect of radiation therapy.

## Competing interests

The author(s) declare that they have no competing interests.

## Authors' contributions

NLS conducted follow-up examinations and contributed to data analysis and drafting the manuscript.

BPS contributed to data analysis and drafting the manuscript.

LG performed assessments of patients in the Rehabilitation Medicine Department and contributed to data analysis and editing the manuscript.

EA performed assessments of patients in the Rehabilitation Medicine Department.

SS contributed to data analysis and editing the manuscript.

RA contributed to the initial design of the study, oversaw the administration of radiation therapy to the patients, conducted follow-up examinations, and contributed to data analysis and editing the manuscript.

JBM contributed to data analysis and editing the manuscript.

KAC oversaw the administration of radiation therapy to the patients, conducted follow-up examinations, and contributed to data analysis and drafting the manuscript.

All authors have read and approved the final version of this manuscript.
